# Understanding the structure of coping strategies in context: a psychometric validation of the Brief-COPE among Colombian adults

**DOI:** 10.1186/s41155-025-00368-9

**Published:** 2025-12-07

**Authors:** Nicolás García-Mejía, Leonidas Castro-Camacho, Judith K. Daniels, Anja F. Ernst, Marieke E. Timmerman, Miriam J. J. Lommen

**Affiliations:** 1 Psychometrics and Statistics, University of Groningen, Groningen, Netherlands; 2 Department of Psychology, Universidad de Los Andes, Bogotá, Colombia; 3 Department of Clinical Psychology and Experimental Psychopathology, University of Groningen, Grote Kruisstraat, 2/1, Groningen, 9712 TS Netherlands; 4 Department Trauma Center, GGZ Drenthe Mental Health Institute, Beilen, Netherlands

**Keywords:** Coping, Validation, Psychometric, Confirmatory factor analysis, Brief-COPE

## Abstract

**Background:**

This study validates the Spanish version of the Brief-COPE in the Colombian context. This tool assesses 14 different coping strategies, including positive coping, planning, emotional support, instrumental support, substance use, and religion, among others. The structural validations of this tool in Latin America, Europe, North America, and Asia yielded heterogeneous results, with validations in Latin America often having limitations in their data analysis methodologies and sample size. This study aims to address these limitations and provide methodologically sound evidence on the structural validity, reliability, and convergent and divergent validity of the instrument for adults in Colombia.

**Methods:**

A total of 762 participants completed the Brief-COPE along with the ERQ, the Wellbeing Index, the HSCL-25, the PCL-C, and the Kessler 6. Categorical Confirmatory Factor Analysis (CFA) was employed to assess the fit of 12 different theory and data-driven models. After identifying the best-fitting model, reliability, divergent, and convergent validity were assessed for the resulting factors.

**Results:**

The best-fitting CFA model for the Brief-COPE had 11 factors: active coping, social support, acceptance, venting, self-distraction, behavioral disengagement, denial, self-blame, humor, religion, and substance use. Substance use, active coping, religion, social support, humor, self-blame, denial, and behavioral disengagement demonstrated good reliability (*Omega* > = .7*)*, whereas the remaining subscales demonstrated insufficient reliability (*Omega* > *.6 and Omega* < .7). Maladaptive coping strategies were found to positively correlate with distress measures, while adaptive strategies exhibited negative correlations, as expected. However, social support and humor presented significant positive associations with PCL-C and HSCL.

**Conclusions:**

This study provides evidence supporting an 11-factor structure for the Brief-COPE in Colombian adults, with most factors demonstrating satisfactory reliability. Researchers should use caution when interpreting subscales with lower reliability. The results also underscore the influence of cultural context on coping patterns, given the heterogeneous factor structures found in other validations. Future studies should recruit more diverse samples to enhance generalizability and further investigate the predictive validity of this adapted tool.

## Introduction

The measurement of coping strategies is essential to understand how individuals face and adapt to challenging and stressful situations. Models of coping, such as Lazarus and Folkman’s (1984) transactional model of stress, highlight how assessment of a situation results in stress and motivates coping. The implementation of different coping strategies has short and long-term positive and negative consequences, some of which are especially important for mental health. For example, different maladaptive coping strategies have been linked to the development and maintenance of mental health and somatic complaints (Day et al., 2022; Güney et al., 2019; Post et al., 2021; Seguin & Roberts, 2017). For example, PTSD is linked to a lack of emotional clarity (Timmer-Murillo et al., 2023), emotional disconnection (Díaz-Tamayo et al., 2022), social withdrawal, wishful thinking, self-criticism, lack of reappraisal skills, and avoidance (Díaz-Tamayo et al., 2022; Morris & Rao, 2013; Seguin & Roberts, 2017; Thompson et al., 2018). The latter is consistently associated to PTSD and anxiety symptom severity and treatment response (Bourdon et al., 2019; Díaz-Tamayo et al., 2022; McNeill & Galovski, 2015; Morris & Rao, 2013; Seguin & Roberts, 2017; Stander et al., 2014). Additionally, strategies such as rumination, suppression, and distraction have been linked with PTSD, depression, anxiety, substance use, and pain (Day et al., 2022; Finstad et al., 2021; Güney et al., 2019; McNeill & Galovski, 2015; Morasco et al., 2013; Post et al., 2021; Stander et al., 2014; Timmer-Murillo et al., 2023). Although these associations are generally accepted, the relationship between coping strategies and mental health problems is nuanced and culture-dependent (Seguin & Roberts, 2017).

Therefore, a valid assessment instrument is essential for advancing research on the complex relationship between coping strategies and mental health in Colombia. While instruments such as the Emotion Regulation Questionnaire (ERQ; Canales Ramírez et al., 2024) and the Difficulties in Emotion Regulation Scale (DERS; Muñoz-Martínez et al., 2016) have been validated for use in Colombia, they focus primarily on emotion-based coping. The DERS covers other relevant coping strategies like goal-focused coping, its emphasis is on emotional regulation, leaving a gap for more comprehensive measures. The Brief—Coping Orientation to Problems Experienced Inventory (Brief-COPE; Carver, 1997) fills this gap while using a comparable item count to the DERS (28 items and 34 items, respectively). It assesses 14 coping strategies, grouped into three higher-order strategies: active coping, planning, instrumental support (grouped into problem-focused), emotional support, positive reframing, acceptance, religion, humor, substance use (grouped into emotion-focused), venting, self-blame, denial, behavioral disengagement, and self-distraction (grouped into avoidance-focused strategies; Cooper et al., 2008).

Carver (1997) originally proposed these 14 subscales, in his initial exploratory factor analysis (EFA), active coping, planning, and positive reframing loaded onto a single factor, while the remaining eleven subscales aligned with their expected structures. Since then, researchers in various cultural settings have suggested alternative models and scoring schemes. In the United States and Europe, for instance, studies consistently find a “positive coping” factor that bundles active coping, planning, and positive reframing, as well as a “social support” factor comprising instrumental support, emotional support, and venting. The subscales of religion, humor, self-blame, and substance use tend to remain consistent with Carver’s original scoring across most samples. The remaining four subscales (acceptance, denial, behavioral disengagement, and self-distraction) have shown much more variability. Some studies group them into a single “avoidance coping” factor, while others treat each as an individual construct. This inconsistency has been reported in a range of research (Baumstarck et al., 2017; Doron et al., 2014; Kapsou et al., 2010; Miyazaki et al., 2008; Monzani et al., 2015; Morán et al., 2010; Muller and Spitz, 2003; Pavlova et al., 2022; Serrano et al., 2021; Su et al., 2015).

In Latin America, the variability among studies is even more pronounced, with several validations eliminating whole items or subscales. For instance, the “instrumental support”subscale appears in five of eight studies (García et al., 2018; Guillén-Díaz-Barriga et al., 2021; Mejorada et al., 2013; Morán et al., 2010; Richard’s et al., 2021), whereas it is absent in the other three (Brasileiro et al., 2016; Reich et al., 2016; Vargas-Manzanares et al., 2010). A similar patterns appear for humor (García et al., 2018; Vargas-Manzanares et al., 2010; Mejorada et al., 2013; Morán et al., 2010) and substance use (García et al., 2018; Guillén-Díaz-Barriga et al., 2021; Morán et al., 2010; Reich et al., 2016) emerging in four of eight validations. “Positive coping” was present in two of the seven validations (Morán et al., 2014; Richard’s et al., 2021), and the remaining original subscales appeared inconsistently across validations. Two main factors explain this heterogeneity. First, different methodological approaches were employed to test the psychometric structure of the Brief-COPE, including the use of Principal Components Analysis (PCA), EFA, CFA, or mixed approaches. Additionally, these studies tend to have small sample sizes ranging from 140 to 300, with only three recruiting over 500 participants (García et al., 2018; Morán et al., 2014; Richard's et al. 2021). Such methodological limitations undermine statistical validity. Second, different populations might present distinct psychometric structures for the Brief-COPE due to diverse learning experiences, preferences in coping, and conceptualizations of mental health and emotions. Therefore, well-founded statistical psychometric research is necessary to identify a culturally adequate psychometric structure for the Spanish version of the Brief-COPE in Colombia.

The only Brief‑COPE validation conducted in Colombia so far used a small sample (*n* = 140) of women with breast cancer (Vargas-Manzanares et al., 2010), limiting both statistical validity and generalizability to a broader Colombian population. Our study aims to provide empirical evidence for the reliability, structural, and criterion validity from a confirmatory framework in a general Colombian sample using an adequate modeling approach and sufficient sample size. Our primary objective is to test various models based on both theoretical structures and previous validation efforts for the Brief-COPE. We will test the following five theoretical models (as presented by Doron et al., 2014): 1.) the original Carver (1997) model, 2.) the Lazarus and Folkman’s (1984) problem-focused and emotion-focused coping model; 3.) Krohne’s (1993) and Roth and Cohen’s (1986) approach and avoidance coping model; 4.) Cooper et al. (2008) problem-focused, emotion-focused, and avoidance coping model; and finally, 5.) Ayers et al. (1996) five-dimensional model includes avoidance, cognitive restructuring, problem-solving, distraction, and support-seeking. In addition, we will test three data-driven models, derived from the most commonly identified factors in validations in Latin America, the US, and Europe (see Table 2). Finally, we also seek to provide evidence for internal reliability for the Brief-COPE and its convergent and divergent validity against measures of emotional distress, emotional regulation, and wellbeing.

## Methods

The current study was preregistered in OSF. The preregistration can be found in the following link: preregistration. Materials, including analysis code, results, and study materials, can be found in the following link: supplementary materials. The dataset for the analysis is not shared due to anonymity concerns.

### Participants

Responses were gathered online from Colombian adults aged 18 or older, who are Spanish speakers and currently reside in Colombia. Our sampling approach was a non-probabilistic snowball and convenience sampling methods; Participants were recruited from the general population through online publicity, university courses, direct contact, and referrals. To secure adequate model convergence, the study’s target sample size was 900 participants. This sample size was decided based on a simulation we performed to test model convergence for a model with 14 first-order factors and five second-order factors, assuming ordinal responses to the observed items. The code, specific assumptions for the model, and simulation results can be found at the following link: simulation.

In total 970 Colombian adults agreed to participate in the study. In our analysis, only participants with complete data for the Brief-COPE and those with missing data in the last two items were included; the latter due to an error in the first wave of data gathering, which did not include said items. This resulted in a sample size of 762. The mean age was 37.5 (*SD* = 13.9; range = 18 to 65), 66.9% were women, and the majority of participants were single (50.3%), married (28.7%), or in cohabitation (11.8%). The majority of the current sample had university education (83.9%), a formal job (58.4%), and declared no ethnic affiliation (96.5%). Additionally, 1.4% were Afro-Colombian, 0.5% were Indigenous, and 0.4% Rom. Finally, only 21 participants (2.7%) reported being forcibly displaced.

### Instruments

#### Validated tool

The Brief-COPE (Carver, 1997; Spanish version by Perczek et al., 2000) is a 28-item tool that assesses 14 coping strategies. Items are responded to on a 4-point Likert scale ranging from 0 (I did not do this at all) to 3 (I did this a lot) with one-point intervals. The complete list of items can be found in Table 1. The Spanish version can be found in the supplementary materials. Copyright owned by Charles Carver protects the Brief-COPE it can be openly and freely use for research and practice: https://www.psy.miami.edu/faculty/ccarver/brief-cope.html.

**Table 1:** Original carver model (1984) of the brief COPE questionnaire

Factor	Item Naming	Item Text
Active Coping	BC02	I’ve been concentrating my efforts on doing something about the situation I’m in
	BC10	I’ve been taking action to try to make the situation better
Informational Support	BC01	I’ve been getting help and advice from other people
	BC28	I’ve been trying to get advice or help from other people about what
Positive Reframing	BC14	I’ve been trying to see it in a different light to make it seem more positive
	BC18	I’ve been looking for something good in what is happening
Planning	BC06	I’ve been trying to come up with a strategy about what to do
	BC26	I’ve been thinking hard about what steps to take
Emotional Support	BC09	I’ve been getting emotional support from others
	BC17	I’ve been getting comfort and understanding from someone
Venting	BC12	I’ve been saying things to let my unpleasant feelings escape
	BC23	I’ve been expressing my negative feelings
Humor	BC07	I’ve been making jokes about it
	BC19	I’ve been making fun of the situation
Acceptance	BC03	I’ve been accepting the reality of the fact that it has happened
	BC21	I’ve been learning to live with it
Religion	BC16	I’ve been trying to find comfort in my religion or spiritual beliefs
	BC20	I’ve been praying or meditating
Self-Blame	BC08	I’ve been criticizing myself
	BC27	I’ve been blaming myself for things that happened
Self-Distraction	BC04	I’ve been turning to work or other activities to take my mind off things
	BC22	I’ve been doing something to think about it less, such as going to movies, watching TV, reading, daydreaming, sleeping, or shopping
Denial	BC05	I’ve been saying to myself, “this isn’t real”
	BC13	I’ve been refusing to believe that it has happened
Substance Use	BC15	I’ve been using alcohol or other drugs to make myself feel better
	BC24	I’ve been using alcohol or other drugs to help me get through it
Behavioral Disengagement	BC11	I’ve been giving up trying to deal with it
	BC25	I’ve been giving up the attempt to cope

#### Convergent and divergent validity tools

To assess nosologically relevant variables, we used the *Emotion Regulation Questionnaire* (ERQ; Gross & John, 2003; Spanish version by Canales Ramírez et al., 2024), a 10-item tool that assesses two emotional regulation strategies: cognitive reappraisal and suppression. The reliability in our sample for the subscales was acceptable (suppression: *alpha* =.73; reappraisal: *alpha* =.70). Additionally, the *Wellbeing Index* (Jovanović et al., 2019; Spanish version by Forjaz et al., 2012) an 8-item tool was used to assess perceived quality of life regarding health, relationships, security, community connections, and future security. Its reliability for our sample was good (*alpha* =.88). To measure anxiety and depression, we used the *Hopkins Symptoms Checklist* (HSCL-25; Derogatis et al., 1974; Spanish version by Clavería et al., 2020); this is a 25-item tool, including 15 items for depression symptoms (*alpha* =.92 in our sample) and 10 items for anxiety symptoms (*alpha* =.91 in our sample). To measure the severity of posttraumatic stress disorder symptoms, we used the *Posttraumatic Symptom Checklist-Civilian Version* (PCL-C; Spanish version by Miles et al., 2008); this is a 17-item tool assessing PTSD in civilian populations; its reliability was good in our sample (*alpha* =.92). Finally, we used the *Kessler 6* (Kessler et al., 2010), a 6-item screening tool that assesses general psychological distress, which showed good reliability in our sample (*alpha* =.88).

### Procedure

Informed consent and self-report questionnaires were presented using the Groningen University license for Qualtrics. All participants provided informed consent before participating in the study. This study was approved by the ethics committee of the Behavioral and Social Sciences Faculty (University of Groningen) under the [PSY-2324-S-0435 Brief-COPE Colombian Psychometric Validation] review code. To ensure the anonymity of responses, identifying information was not collected (including names, last names, emails, telephone numbers, and IP addresses). Other sensitive information, such as ethnicity, age, gender, education, and mental health self-reports, is stored on a secure hard drive managed by the University of Groningen, and individual data is only accessible to the main researchers.

### Analysis

#### Missing data

An error during the first wave of data collection which caused 56% (*n* = 426) of participants to miss items BC27 and BC28 on the Brief‑COPE. Because the missingness was attributable to a procedural mistake rather than participant characteristics, we treated it as missing completely at random. To handle this, we identified predictors of missingness by regressing a missing data indicator on demographic variables and theoretically related items from the Brief-COPE using logistic regression. After determining the predictors of missingness, we imputed 100 datasets with values for BC27 and BC28, based on significant demographic predictors and all Brief-COPE items. Following the recommendations of Enders and Little (2022). This “agnostic” approach enhances the estimation of plausible values, as it utilizes the full variance–covariance matrix of the items to predict missing values. The imputation was performed employing the proportional odds regression method since the responses for these items are categorical and ordered (Enders & Little, 2022). The imputation was conducted using the “mice” (Van Buuren & Groothuis-Oudshoorn, 2006) package in R (R Core Team, 2022).

#### Structural validity

We tested five theory-based models that included the originally proposed 14 factors. Additionally, we tested four model variations that excluded the original 14 first-order factors, and three data-driven models, for a total of 12 models. For definitions of all models, please refer to Table 2. These models were estimated using CFA with Diagonally Weighted Least Squares (DWLS; DiStefano & Morgan, 2014). We chose to use DWLS estimation as it is the preferred estimator for ordered categorical data such as the Brief‑COPE’s four‑point Likert items (Forero et al., 2009). To ensure model identification when including factors with only two items (e.g., models that include the 14 first-order factors of the Brief-COPE), we constrained the factor loadings of both items to be equal, in line with the solution from Grieder et al. (2023) for two-item factor model identification.

**Table 2:** Structural validity models tested

Models	Number of Factors		Factor Descriptions
Theory-based models	Second Order	First Order	
Carver Original Model		14	Planning, active coping, instrumental support, acceptance, positive reframing, emotional support, behavioral disengagement, self-distraction, self-blame, humor, denial, religion, venting, and substance use
Problem and Emotion Focused	2	14	Problem-focused: planning, active coping, instrumental support Emotion-focused: acceptance, positive reframing, emotional support, behavioral disengagement, self-distraction, self-blame, humor, denial, religion, venting, and substance use
Problem and Emotion Focused (No 2nd order factor)		2	Problem-focused Emotion-focused
Approach and Avoidance Coping	2	14	Approach coping: acceptance, active coping, positive reframing, planning, humor, religion, instrumental support, emotional support Avoidance coping: behavioral disengagement, self-distraction, self-blame, denial, venting, substance use
Approach and Avoidance Coping (No 2nd order factor)		2	Approach coping Avoidance coping
Problem, Emotion, and Avoidance Focused	3	14	Problem-focused: planning, active coping, instrumental support Emotion-focused: acceptance, positive reframing, emotional support, self-distraction, humor, and religion Avoidance Coping: behavioral disengagement, self-blame, denial, venting, and substance use
Problem, Emotion, and Avoidance Focused Coping (No 2nd order factor)		3	Problem-focused Emotion-focused Avoidance-focused
Five factors Ayers et al. (1996)	5	14	Avoidance: Behavioral disengagement, self-blame, denial, substance use Cognitive restructuring: Humor, Positive Reframing, Acceptance Problem Solving: Active Coping, Planning Distraction: Self-Distraction, Venting Support Seeking: Instrumental Support, Emotional Support, Religion
Five factors Ayers et al. (1996) (No 2nd order factor)		5	Avoidance Cognitive restructuring Problem-solving Distraction Support seeking
Data-driven models	Second Order	First Order	Factor Descriptions
Common Factors #1		11	Factor 1: active coping, positive reframing, planning Factor 2: emotional, instrumental support Individual coping strategies: venting, humor, acceptance, religion, self-blame, self-distraction, Denial, Substance Use, Behavioral Disengagement
Common Factors #2		9	Factor 1: active coping, positive reframing, planning, humor Factor 2: emotional, instrumental support, venting Individual coping strategies: acceptance, religion, self-blame, self-distraction, Denial, Substance Use, Behavioral Disengagement
Common Factors #3		3	Factor 1: active coping, positive reframing, planning, humor Factor 2: emotional, instrumental support, venting, acceptance, religion Factor 3: self-blame, self-distraction, denial, substance use, behavioral disengagement

Furthermore, according to the T-rule, all models were overidentified (Koğar & Yilmaz Koğar, 2015), indicating that we had sufficient degrees of freedom in all models for accurate estimation (T-rule calculations can be found in additional materials). All analyses were conducted in R (R Core Team, 2022). We estimated each proposed model across the 100 imputed datasets and pooled the results following Rubin’s rules (Enders & Little, 2022) using the “lavaan.mi” package (Jorgensen et al., 2012), which was developed for structural equation modeling (SEM) with multiple imputations.

#### Model fit and model selection criteria

After model estimation, we assessed the fit criteria using the thresholds suggested by Schreiber et al (2006). A model is considered to have adequate fit if the following conditions are met: comparative fit index (CFI) ≥ 0.95, square error of approximation (RMSEA) ≤ 0.06, and standardized root mean square residual (SRMR) ≤ 0.08. Models that do not converge were deemed invalid and discarded. Next, we selected the models that achieved an adequate fit and compared them based on parsimony, opting for those with fewer factors, as no comparative indices, such as Bayesian Information Criterion (BIC) or Akaike Information Criterion (AIC), are available for the DWLS estimation (Burnham & Anderson, 2004).

Additionally, if a model that includes second-order factors was chosen, it was compared with a nested model that did not. For example, the Ayers 5-factor model, which includes 14 first-order factors, would be compared to the 5-factor model without the 14 first-order factors (see Table 2). If there are no significant differences in fit between these two models, the more parsimonious model is favored. Model equivalence was established based on the criteria set by Chen (2007), who proposed the following fit differences as indicators of worst fit: ΔCFI < −0.01, ΔRMSEA > 0.015, and ΔSRMR > 0.03. This comparison is valid since the first-order factor model is nested within the second-order factor model.

#### Reliability

For the selected model, the reliability of the test and its subscales was estimated using McDonald’s *Omega* and Ordinal Cronbach’s *Alpha*; we consider *Omega* the best reliability estimation since it does not assume *tau* equivalence, but we keep *Alpha* for comparison purposes (Hayes & Coutts, 2020). Reliability was estimated by using the “compRelSEM” function from the “lavaan.mi” package, which pools reliability results from the different models estimated on the multiple imputed datasets (Jorgensen et al., 2012). This method measures *Omega,* making use of latent scores and *Alpha* based on the polychoric correlation matrix.

#### Convergent and divergent validity

To evaluate the convergent and divergent validity of the Brief‑COPE subscales that emerged in our best‑fitting model, we examined their relationships with a range of mental‑health indicators (anxiety, depression, PTSD), measures of emotional regulation (positive reappraisal, suppression), and overall wellbeing. Although exploratory, we formulated the following expectations: Positive reappraisal should correlate positively with Brief‑COPE strategies that reflect adaptive coping—such as humor, positive reframing, and acceptance. Suppression is expected to be linked to maladaptive Brief‑COPE behaviors (behavioral disengagement, denial, self‑distraction). Problem‑focused coping and support‑seeking should show positive associations with wellbeing and negative correlations with PTSD, depression, anxiety, and general distress. In contrast, avoidance and distraction strategies are anticipated to correlate positively with PTSD, depression, anxiety, and general distress, while showing a negative association with wellbeing. We computed Pearson correlations to test the relationship of the Brief-COPE identified subscales with mental health indicators, wellbeing, and emotion regulation strategies. Bivariate scatterplots were explored to assess non-linear relationships and assess the need for other correlation measures, such as Spearman’s *rho* (Schober et al., 2018).

## Results

### Multiple imputation

Exploratory analyses of missingness revealed that age and marital status significantly predicted non‑response on items BC27 and BC28. This pattern likely reflects a procedural errors: after the data‑collection protocol was corrected, most respondents were university students (generally younger and more often single), whereas earlier respondents tended to be employed adults who were married. To account for this, we included age and civil status as predictors in the imputation procedure. The imputation was successfully implemented and generated 100 imputed datasets with plausible values for items BC27 and BC28. The distribution of imputed variables is included in supplementary materials.

### Strucutral validity analysis

Of 12 models tested, we chose the common factor model #1 (Table 3), as the best-fitting model of the Brief-COPE in this sample, since it was the only model that showed an adequate fit. Other competing theoretical models did not achieve adequate model fit or did not converge adequately. Based on these results, we select the common factors #1 model as the best-fitting model for the Brief-COPE in this sample. This model comprises 11 factors (Table 4), an Active Coping factor, which is composed of the items from the original Carver subscales of active coping, positive reframing, and planning. Social Support comprised the original Brief-COPE items for emotional and instrumental support. The other nine factors come directly from the original subscales: Venting, Humor, Acceptance, Religion, Self-blame, Self-distraction, Denial, Substance Use, and Behavioral Disengagement. All items included in the scale have a factor loading higher than.6 (Table 4).

**Table 3:** Confirmatory factor analysis models fit indices

Model Names	*CFI*	*TLI*	*RMSEA*	*SRMR*	Convergence
Common factors #1	**.974**	**.968**	**.055**	**.059**	100%
Common factors #2	.881	.860	.114	.110	100%
Common factors #3	.826	.808	.133	.139	100%
Carver original	.944	.917	.089	.082	0%
Lazarus & Folkman	.809	.791	.141	.155	0%
L&F—no 1 st order	.596	.558	.205	.230	92%
Roth & Cohen	.841	.825	.129	.135	0%
R&C—no 1 st order	.646	.612	.192	.186	100%
Carver (1997)	.848	.832	.126	.137	0%
C. (1997)—no 1 st order	.651	.614	.192	.187	100%
5 factors Ayers	.887	.872	.110	.117	0%
5 F A.—no 1 st order	.800	.774	.147	.152	100%

**Table 4:** Confirmatory factor analysis common factors #1 results

Subscale/Item	Item Text	*Factor Loadings*
Active Coping		
BC26	I’ve been thinking hard about what steps to take	.61
BC06	I’ve been trying to come up with a strategy about what to do	.67
BC02	I’ve been concentrating my efforts on doing something about the situation I’m in	.68
BC14	I’ve been trying to see it in a different light, to make it seem more positive	.75
BC10	I’ve been taking action to try to make the situation better	.77
BC18	I’ve been looking for something good in what is happening	.84
Social Support		
BC01	I’ve been getting help and advice from other people	.70
BC28	I’ve been trying to get advice or help from other people about what to do	.85
BC09	I’ve been getting emotional support from others	.90
BC17	I’ve been getting comfort and understanding from someone	.92
Acceptance		
BC03 (a)	I’ve been accepting the reality of the fact that it has happened	.67
BC21 (a)	I’ve been learning to live with it	.67
Venting		
BC12 (b)	I’ve been saying things to let my unpleasant feelings escape	.71
BC23 (b)	I’ve been expressing my negative feelings	.71
Self-Distraction		
BC04 (c)	I’ve been turning to work or other activities to take my mind off things	.72
BC22 (c)	I’ve been doing something to think about it less, such as going to movies, watching TV, reading, daydreaming, sleeping, or shopping	.72
Behavioral Disengagement		
BC11 (d)	I’ve been giving up trying to deal with it	.75
BC25 (d)	I’ve been giving up the attempt to cope	.75
Denial		
BC05 (e)	I’ve been saying to myself “this isn’t real”	.79
BC13 (e)	I’ve been refusing to believe that it has happened	.79
Self-blame		
BC08 (f)	I’ve been criticizing myself	.85
BC27 (f)	I’ve been blaming myself for things that happened	.85
Humor		
BC07 (g)	I’ve been saying to myself, “This isn’t real”	.92
BC19 (g)	I’ve been refusing to believe that it has happened	.92
Religion		
BC16 (h)	I’ve been trying to find comfort in my religion or spiritual beliefs	.93
BC20 (h)	I’ve been praying or meditating	.93
Substance Use		
BC15 (i)	I’ve been using alcohol or other drugs to make myself feel better	.97
BC24 (i)	I’ve been using alcohol or other drugs to help me get through it	.97

Items with (‘letter’) at its side indicate that factor loadings are fixed equal to its factor pair. For example, BC11 (i) and BC25 (i) both belong to Behavioral Disengagement and have their factor loading fixed to be equal for the factor they belong to (i.e., the factor loading is.75 for both items)

### Reliability

Internal reliability measures (Table 5) were generally consistent for *Alpha* and *Omega* estimates, except for active coping, where *Omega* (.92) was .08 higher than *Alpha* (.84). Nevertheless, this difference doesn’t change the qualitative interpretation of the subscale consistency, which has good reliability. Other subscales with good reliability (*Omega* >.80) were social support, humor, religion, self-blame, and substance Use. Denial and behavioral disengagement had acceptable reliability (*Omega* >.70). Finally, Self-distraction, venting, and acceptance had insufficient reliability (*Omega* >.60 and *Omega* < =.7).

**Table 5:** Brief-COPE subscales reliability

Brief-COPE Subscales	*Alpha*	*Omega*
Substance Use	0.97	0.97
Religion	0.93	0.93
Active Coping	0.84	0.92
Social Support	0.90	0.91
Humor	0.91	0.91
Self-Blame	0.84	0.84
Denial	0.76	0.76
Behavioral Disengagement	0.72	0.72
Self-Distraction	0.68	0.68
Venting	0.67	0.67
Acceptance	0.62	0.62

### Validity with other measures

Table 6 presents the matrix of correlations between the identified Brief-COPE subscales and ERQ, Kessler 6, HSCL, PCL-C, and Wellbeing. Active coping, and the coping strategies generally considered to be maladaptive (such as self-distraction, denial, substance use, behavioral disengagement, and self-blame) showed the hypothesized associations with the HSCL anxiety and depression, PCL-C, ERQ, and wellbeing. On the other hand, social support and humor had correlations contrary to what was hypothesized, showing positive correlations with PCL-C, HSCL-depression, and HSCL-anxiety. Finally, acceptance and religion had no significant relationships with PCL-C, HSCL-depression, and HSCL-anxiety.

**Table 6:** Brief-COPE subscales correlations with other psychological indicators

	PCL-C	ERQ Suppression	ERQ Reappraisal	HSCL Depression	HSCL Anxiety	Kessler-6	Wellbeing
Active Coping	**-.13**	**-.23**	**.35**	**-.17**	**-.09**	**-.25**	**.31**
Social Support	**.11**	**-.26**	.07	**.12**	**.14**	.07	.05
Venting	**.20**	**-.20**	.05	**.20**	**.20**	**.14**	-.01
Humor	**.16**	.01	.05	**.10**	**.12**	**.11**	-.03
Acceptance	-.02	-.07	**.14**	-.02	-.07	**-.08**	**.11**
Religion	-.03	**-.10**	**.21**	-.03	.02	**-.12**	**.25**
Self-distraction	**.38**	**.13**	**.12**	**.37**	**.29**	**.29**	**-.14**
Denial	**.33**	**.15**	**.09**	**.26**	**.32**	**.25**	**-.10**
Substance Use	**.31**	**.11**	-.04	**.27**	**.26**	**.26**	**-.16**
Behavioral Disengagement	**.39**	**.17**	-.06	**.37**	**.31**	**.41**	**-.28**
Self-Blame	**.49**	**.13**	**-.11**	**.53**	**.40**	**.47**	**-.37**

Correlations in bold are significant *P* < 0.05

## Discussion

### General results

Our study aimed to study the psychometric properties, especially structural validity, of the Brief-COPE in a general Colombian sample using an adequate sample size and methodologically sound CFA. We found the best-fitting model to have 11 factors. The factor active coping includes the original active coping, positive reframing, and planning items. The factor social support includes original emotional and instrumental support items. The other nine factors are the originally proposed factors: venting, humor, acceptance, religion, self-distraction, substance use, behavioral disengagement, self-blame, and denial, which are each composed of two items. We found good reliability for six subscales, acceptable reliability for two, and inadequate reliability for three. Finally, we found evidence of convergent and divergent validity with distress measures, coping strategies, and wellbeing. Although the structure closely resembles the original Carver (1997) model, the variability of structural results across different samples merits further discussion.

### Structural validity

The 11-factor structure identified in our study closely reproduces the original Brief-COPE EFA. Carver’s (1997) EFA groups active coping, positive reframing, and planning as a factor, emotional and instrumental support are grouped together, and self-blame and denial are also grouped together. The other seven proposed factors resulted as initially hypothesized by Carver (1997). The factor structure supported by our study reproduces all the mentioned factors, except for the grouping of self-blame and denial, which are separated subscales in our analyses. Furthermore, there are five confirmatory validation studies of the Brief-COPE; Three of these validations supported the original 14-factor structure (García et al., 2018; Monzani et al., 2015; Muller & Spitz, 2003); one supported the 5-second order factor by Ayers et al. (1996) model (Doron et al., 2014), and one resorted to using EFA since the confirmatory model showed bad fit (Su et al., 2015). In our study, the 14-factor model did not converge. We attribute this failure to the use of DWLS estimation, which is designed for ordinal indicators but can struggle with highly complex models. This is not the case for these confirmatory validations, which use Maximum Likelihood (Doron et al., 2014; Monzani et al., 2015; Muller & Spitz, 2003; Su et al., 2015) and robust Maximum Likelihood (MLR; García et al., 2018); these estimation methods have an easier time with these complex models. Nevertheless, DWLS better reflects the requirements of the Likert-type items and thus provides a more appropriate assessment of the Brief-COPE structure.

Compared to the other Colombian validation (Vargas-Manzanares et al., 2010), the structures differ considerably. These differences might be explained by sample composition and methodological reasons. First, we gather data from the general population compared to women with breast cancer in the Vargas-Manzanares et al. (2010) validation. Second, the Vargas-Manzanares et al. (2010) validation had a small sample size (*n* = 140) and used a PCA and EFA approach that did not converge, which raises doubt on the adequacy of the model. Other Latin American validations also differ importantly from our identified structure; various studies drop several items due to their use of EFA or PCA and small sample sizes (Brasileiro et al., 2016; Guillén-Díaz-Barriga et al., 2021; Mejorada et al., 2013; Reich et al., 2016; Richard’s et al., 2021). There are two methodological exceptions; one is the Chilean validation (García et al., 2018) that replicates the theoretical 14 factors with a sample size of 1847, although it used ML to estimate their models (thereby assuming items are continuous). The other one is the Brazilian validation, which shows the same active coping in a social support structure, but groups religion, self-distraction, and denial into one factor, resulting in a total of 8-factors structure (using EFA and a sample size of 899 participants).

When compared to validations in Latin America, the US, and Europe, the active coping factor (including active coping, positive reappraisal, and planning items) and the social support factor (including instrumental and emotional support) are consistent for validations in Argentina, Brazil, Mexico, the US, France, Russia, and Greece (Carver, 1997; Doron et al., 2014; Kapsou et al., 2010; Miyazaki et al., 2008; Morán et al., 2010, 2014; Pavlova et al., 2022; Richard’s et al., 2021; Serrano et al., 2021). Other scales like humor, acceptance, and religion tend to appear separated (Carver, 1997; García et al., 2018; Kapsou et al., 2010; Mejorada et al., 2013; Monzani et al., 2015; Morán et al., 2010, 2014; Muller & Spitz, 2003; Pavlova et al., 2022; Serrano et al., 2021; Vargas-Manzanares et al., 2010). Moreover, “negative” coping strategies such as self-blame, self-distraction, denial, substance use, and behavioral disengagement sometimes group together at a first-factor level (Brasileiro et al., 2016; Carver, 1997; Guillén-Díaz-Barriga et al., 2021; Morán et al., 2014; Vargas-Manzanares et al., 2010), sometimes are grouped on a second-order factor (Doron et al., 2014; Morán et al., 2010; Serrano et al., 2021), or remain separate as a first order factor (García et al., 2018; Kapsou et al., 2010; Miyazaki et al., 2008; Monzani et al., 2015; Muller & Spitz, 2003; Reich et al., 2016). In general, active coping strategies and social support tend to factor together across regions; in some cases, strategies like humor, religion, and acceptance are included in active coping, and the “negative” coping strategy factor structure varies widely across cultures.

### Reliability results

Regarding the reliability of our 11 subscales, most had good reliability, and three showed insufficient reliability. One of the explanations for this issue is that we are using only two items in each of the inadequate reliability subscales (venting, self-distraction, and acceptance). Nevertheless, other subscales like self-blame, religion, humor, and substance use are composed of only two items and show good reliability. These differences might be explained by item content. For acceptance, items seem to be referring to two processes of acceptance: item BC03 refers to “accepting the reality of what happened,” and BC21 refers to “learning to live with it”, with BC21 reflecting a different theme in the acceptance construct. For self-distraction, the distraction activities refer to different domains; BC04 suggests distraction with work, and BC22 suggests distraction with leisure activities (like watching TV). Denial showed acceptable reliability also presenting a difference in the theme of items, with BC05 referring to a thought “this isn’t real” and BC13 referring to a stronger belief about “refusing to believe it happened”. This differentiation in the themes of the items might explain the lack of reliability.

On the other hand, venting might have translation inconsistencies. Venting BC23 uses the Spanish word “pensamientos” (thoughts) instead of “sentimientos” (feelings), the latter being the original English term, which is also used in item BC12. Behavioral disengagement showed acceptable reliability and showed translation inconsistencies. The English version uses “I’ve been giving up” for both BC11 and BC25 items, but in the Spanish version, the BC11 item uses the term “me di por vencido” (I’ve given up), and BC25 uses “dejé de hacerle frente” (I stopped facing it). These inconsistencies might explain the reduced reliability. Nevertheless, it is also important to mention that Brief-COPE validations consistently show low reliability for the two-item subscales (Brasileiro et al., 2016; Carver, 1997; Doron et al., 2014; García et al., 2018; Kapsou et al., 2010; Mejorada et al., 2013; Richard’s et al., 2021).

### Validity with other measures

The maladaptive coping subscales that we examined (such as self-distraction, denial, substance use, behavioral disengagement, and self-blame) displayed the expected positive relationships with measures of distress such as PTSD, depression, and anxiety. These findings are consistent with earlier validations (Baumstarck et al., 2017; Doron et al., 2014; Kapsou et al., 2010; Muller & Spitz, 2003). However, their associations with the Emotion Regulation Questionnaire’s reappraisal scale were less straightforward. Denial and self‑distraction showed significant positive correlations with reappraisal, while substance use and behavioral disengagement did not differ significantly from zero, and self‑blame was negatively related to reappraisal. Which suggests a complex interplay between coping strategies. Venting, which we omitted from the initial set since it can be conceptualized as opposed to suppression, but also as a way of unhealthy emotional coping, in our case, venting has positive correlations with distress measures. Which also aligns with longitudinal work showing similar links for depression and anxiety (Marr et al., 2022; Trần et al., 2023).

In contrast, the subscales generally regarded as adaptive yielded mixed results. Active coping behaves as expected, showing negative correlations with distress measures (PTSD, depression, and anxiety) and suppression, and as expected positive correlations with wellbeing and reappraisal. These relationships with active coping have been found in other validations with measures of wellbeing in Chile (García et al., 2018), perceived stress, general mental health, and trait anxiety in France (Baumstarck et al., 2017; Doron et al., 2014; Muller & Spitz, 2003). By contrast, the anticipated negative relationship between social support and distress did not hold; instead, a positive correlation was observed. This may reflect reverse causality: individuals experiencing higher distress are more likely to seek out or report greater social support Similar negative associations between emotional and instrumental support with distress, perceived stress, and mental health found in other validations support this explanation (Baumstarck et al., 2017; Doron et al., 2014; Kapsou et al., 2010; Muller & Spitz, 2003). Furthermore, Su et al. (2015) validation showed a positive correlation between perceived social support and Brief-COPE social support, which is nosologically coherent.

The humor subscale showed positive, albeit small, correlations with distress measures, contrary to our hypothesized association. This might be due to the wording of items that assess humor in a general way rather than differentiated types of humor, such as self-defeating or affiliative humor, which have differentiated effects on mental health (Bedoya Cardona et al., 2023; Schermer et al., 2022; Schneider et al., 2018). Finally, acceptance had positive, although small, correlations with reappraisal and wellbeing. No significant relationships existed between distress measures and acceptance, but these correlations might be attenuated due to its low reliability (Fan, 2003).

### Limitations

Due to a mistake in the tool presentation in Qualtrics, our dataset presented with missing data for two items included in the subscales of social support and self-blame. However, we have strong reason to believe that our data is missing at random and that our multiple imputation procedure is valid. However, this might have impacted the convergence of the models. We found that no model converged in any imputed datasets when models included 14 first-order factor indicators. This is surprising since our simulation showed that these models should have converged 86% to 91% of the time with a sample size of 700 to 800. These problems can be explained by an issue with the estimation method that requires large sample sizes to achieve convergence. However, the DWLS is considered an adequate method to deal with this problem (Forero et al., 2009).

During the confirmatory factor‑analysis phase we encountered several non‑convergence problems that were mainly attributable to Heywood cases (negative variances and correlations or factor loadings higher than 1); While a variety of corrective procedures could resolve such errors, implementing these adjustments across 100 separate data sets proved impractical. A further limitation concerns the composition of our sample, which restricts the extent to which our findings can be generalized. Our sample was mainly composed of highly educated (university or higher) single women with no declared ethnic affiliation; this reflects a bias of our data-gathering process through snowball and online publicity. This limits the generalization of our results and signals the necessity to diversify the samples in which this tool is validated in future research. For example, including participants with lower levels of education and increasing the participation of men who might use different coping strategies due to differences in socialization, development, and gender roles.

### Utilization and future research

One of the main issues with the Brief-COPE is its scoring; with multiple validations showing different structures, the way scales and subscales are scored varies from validation to validation. Based on our current study, we consider that the 11 subscales identified can be used to screen for preferred coping strategies and inform research in the Colombian population. Nevertheless, we suggest caution when using subscales with low reliability (*Omega* between 0.61 and 0.70), like self-distraction, venting, and acceptance. Specifically, in the case of research, it is recommended that the reliability of the subscales be assessed in each research sample to determine how reliable the subscale composite is for data analysis. For assessment and clinical use, we suggest that the Brief-COPE be used as a screening tool to guide clinical decision-making and suggest further exploration into consultants’ underdeveloped positive coping strategies and the use of negative coping strategies. Finally, if the objective is to measure specific characteristics of coping (e.g., such as different kinds of humor use) researchers should consider other options, as the Brief-COPE is designed for screening and practical measurement purposes.

In terms of future research, expanding the sample to address the current limitations regarding generalizability to the Colombian population is important. Including participants with different levels of education and diverse ethnicities can help identify further issues with the measurement and ensure that the tool is generalizable beyond highly educated Colombians with limited ethnic affiliations. Other questions remain about the current tool; its invariance across genders, ethnicities, age ranges, physical health, and mental health problems still needs to be examined, which is required to make direct comparisons between groups using raw scores. Its predictive validity in our target population is still unknown; specifically, how the Brief-COPE can predict future outcomes of mental health and wellbeing is essential to assess its utility in clinical and health settings. Finally, future studies should correct and assess the effect of changing the inconsistent terminology in items composing the low-reliability subscales to improve their internal consistency.

## Conclusions

This study aimed to provide evidence on the structural validity, reliability, and convergent and divergent validity of the Spanish version of the Brief-COPE for Colombian adults. Using an appropriate sample size and categorical CFA, we identified that the best-fitting model for the Brief-COPE in our Colombian adult sample is an 11-factor structure with the following factors: active coping, social support, acceptance, venting, self-distraction, behavioral disengagement, denial, self-blame, humor, religion, and substance use. We found good reliability for active coping, social support, humor, self-blame, religion, behavioral disengagement, denial, and substance use; however, we suggest caution when interpreting results from the remaining scales, which showed low reliability. The convergent and divergent validity showed coherent evidence with the expected nosological relationships. We emphasize the consideration of cultural differences when interpreting and applying the Brief-COPE, as its structure appears to be highly dependent on the validation setting. Finally, we emphasize the need to expand the current validation to more diverse samples, including different ethnic groups, testing with participants with varying levels of education, and including more men in the validation efforts to better understand how the Brief-COPE structure functions in the Colombian population.

## Data Availability

The materials, including the Spanish version of the Brief-COPE, R scripts for data preparation, imputation, analysis, and summary of results, can be found in the following link: supplementary materials. The data on which the study analyses were run is not publicly available.

## Abbreviations

AIC: Akaike Information Criterion
BIC: Bayesian Information Criterion
Brief-COPE: Brief—Coping Orientation to Problems Experienced Inventory
CFA: Confirmatory Factor Analysis
CFI: Comparative Fit Index
DERS: Difficulties in Emotion Regulation
DWLS: Diagonally Weighted Least Squares
ERQ: Emotion Regulation Questionnaire
EFA: Exploratory Factor Analysis
HSCL-25: Hopkins Symptoms Checklist
PCA: Principal Components Analysis
PCL-C: Posttraumatic Symptom Checklist-Civilian Version
PTSD: Posttraumatic Stress Disorder
RMSEA: Root Mean Square Error of Approximation
SEM: Structural Equation Modeling
SRMR: Standardized Root Mean Square Residual
